# Comparing the Patients with or without Inner Ear Malformations in Terms of Intra and Postoperative Complications of Cochlear Implantation

**DOI:** 10.22038/IJORL.2023.71581.3433

**Published:** 2023-09

**Authors:** Adib Molaei Sisakht, Seyed Basir Hashemi, Leila Monshizadeh

**Affiliations:** 1 *Department of Otorhinolaryngology-Head and Neck Surgery, School of Medicine, Shiraz University of Medical Sciences, Shiraz, Iran.*; 2 *Otolaryngology Research Center,* *Department of Otolaryngology, Shiras University of Medical Sciences, Shiraz- Iran.*

**Keywords:** Cochlear implantation, Inner ear malformation, Complications, Gusher

## Abstract

**Introduction:**

Since 20% of the patients with sensorineural hearing loss have confirmed radiographically inner ear malformation, this study aimed to compare the incidence rate of intra and postoperative complications of cochlear implantation amongst the patients with or without inner ear malformations.

**Materials and Methods:**

In this retrospective study, we evaluated the medical record of 954 patients who had undergone cochlear implantation. Seventeen patients had inner ear malformations and were selected as the case group, and 25 patients with normal inner ear were selected as the control group. Patient’s information; including intraoperative complications, post-operative complications and neural response telemetry (NRT) immediately after the surgery were recorded. Finally, the collected data were analyzed, using the SPSS software, version 21.

**Results:**

According to the CT-scan findings, the most observed internal ear abnormality was the temporal bone Mondini in 8 patients (47%), and the next inline was the common cavity at a frequency of (23.52%). Cerebrospinal fluid (CSF) gusher was detected in 11 patients (64.7%) of the case group, which was significantly higher than the other group. This includes mild CSF gusher in 7 cases (41.17%) and severe CSF gusher in 5 cases (29.41%). Interestingly, no significant post-operative complications were observed in either group, minor side effects were limited and not significantly different.

**Conclusion:**

In conclusion, despite the limitations of cochlear implantation surgery amongst patients with inner ear malformation with potential risk of early or late complications, it can still be recommended as an appropriate procedure to acquire hearing as well as auditory and speech perception.

## Introduction

Cochlear Implants (CI) surgery is an effective way to restore hearing in patients with profound hearing loss and those who do not benefit from hearing aids (1). Congenital deafness occurs in approximately one in 1000 children, with at least 60% of it to be hereditary (2,3). It is estimated that 70% of the hereditary cases is non-syndromic (4). Meanwhile, around 20% of the patients with sensorineural hearing loss have inner ear abnormalities (5). The most common abnormality of the inner ear is a large vestibular aqueduct (LVA) (1). Furthermore, other cochlear abnormalities include labyrinthine aplasia, cochlear aplasia, common cavity, cystic cochlea vestibular anomaly (incomplete partition type I), cochlear hypoplasia, and Mondini anomaly (incomplete partition type II) (6). 

There might be particular challenges in the implantation of malformed cochleae when placing a cochlear implant. Generally, cochlear implants complications are divided into major and a minor category. Major ones might require reoperation or hospitalization, including facial nerve stimulation, electrode removal, implant replacement, severe infection, improper electrode placement, or device failure. Whereas, minor complications are tinnitus, postoperative dizziness, taste disorders, mild infections, and transient facial nerve paralysis (7). Cerebrospinal fluid (CSF) gusher is a common complication, affecting approximately 40% of patients with internal ear anomalies, which is associated with meningitis (4, 8). The biggest problem with CSF leakage is its unpredictability until the moment when the cochleostomy is performed. In case of CSF leaks intraoperative management of the leakage may be difficult, and inadequate interventions may lead to severe postoperative intracranial infectious complications. Different studies indicate that about a quarter of cochlear implantation surgeries in patients with inner ear malformation are complicated because of the severity of malformed anatomy or CSF leaks (gusher), but major complications are rare, and the rate of revision surgeries is comparable to that seen among patients who have normal inner ear anatomy (4,8).

Since the inner ear anomalies might be a positive predictor of intra or post-operative complications, the aim of this study was to investigate the incidence rate of cochlear implant complications amongst patients with or without inner ear malformations. 

## Materials and Methods

After obtaining the ethic code of (IR.SUMS.med.REC.1394.09) from our local ethics committee, the present retrospective study was carried out amongst patients with pre-lingual deafness, who underwent cochlear implant surgery. Also, all the participants' parents signed a general informed written consent form to permit using the data of their medical records with consideration of their privacy. We evaluated the medical record of 954 patients who had undergone cochlear implantation before. From the investigated patients, 17 had anomalies of the inner ear and were considered as the case group. The temporal bone CT-scan of the case group was examined by a radiologist, and the type of anomaly was determined. According to the sample size of similar studies (9,10), we selected 25 cochlear implanted patients with normal CT-scan of the temporal bone as a control group. Patients in the case and control groups were matched with respect to age, sex, and the duration of operation. All the patients with accompanying disabilities were excluded from the study. 

Patient’s demographic information was collected from their medical files. The type of anomaly for the case group was also investigated and recorded. Complications during surgery, such as cerebrospinal fluid leakage, lack of proper electrode placement, facial nerve dehiscence, and recording the auditory nerve response immediately after Neural Response Telemetry (NRT) were also collected from the patient’s records. Any major or minor postoperative complications, such as mild prosthesis infection, middle ear infection, facial nerve palsy, facial nerve stimulation, slight hematoma, dizziness, electrode removal, severe prosthetic infection, meningitis, complete facial nerve palsy, extensive hematoma, and prosthetic dislocation, were noted and recorded for at least one-year follow-up by collecting the information from their medical records or getting in touch with a patient via a telephone call. After data collection, the group's complications were compared, using Statistical Package for Social Sciences, version 21(SPSS- 21). According to non-normal distribution of the variables, Mann-Whitney test was carried out to compare the two groups with respect to age, intra and post-operative complications. The statistical significance was set at P<0.05. 

## Results

In total, 42 pre-lingual patients with severe to profound sensorineural hearing loss who underwent cochlear implant surgery were examined. In the case group, mean age of participants was 2.83±0.63 years old, and 2.88±0.78 years old for the control group. Out of which, 17 patients in the case group consisted of 11 girls and 6 boys, and the 25 participants in the control group comprised of 13 girls and 12 boys. The descriptive statistics of the patients in the case and control groups (the patients with/without inner ear anomaly), are exhibited in Table 1.

**Table 1:** The descriptive statistics of the patients with/without inner ear anomaly

**Percent**	**N**	**Patients with normal inner ear**		
80	20	No		Intra operative complications
20	5	Gusher (Very mild)		
100	25	Total		
88	22	No		Post-operative complications
12	3	Minor complications		
100	25	Total		
**Patients with inner ear anomaly**				
47	8	Mondini	Anomaly type	
41.2	7	Common cavity		
5.9	1	Wide IAC		
5.9	1	Narrow IAC		
35.3	6	No	Intra operative complications	
64.7	11	Gusher		
29.4	12	No	Post-operative complications	
70.6	5	Minor complications		
100	17	Total		

Based on the temporal bone CT-scan findings of the case group, most of the inner ear anomalies included Mondini and Common cavity. The two other anomalies (narrow and wide IAC) were observed in 2 patients. 

Crebrospinal fluid (CSF) gusher was observed in 11 patients of the case group. It included seven mild cases and four severe cases. The gusher is considered mild if it comes out slowly. It is severe when it comes out with pressure and fills the middle ear and mastoid spaces (7,8). Mild CSF gusher was occurred in some of the patients with normal inner ear (5 out of 25 patients). Since it is recommended to wait until the gusher stops completely and its pressure become decreased, we implemented the same method during the surgery. After that, it was possible to place the prosthesis and seal its surrounding with soft tissue (7). Due to non-normal distribution of the variables with respect to age, intra operative complications and post-operative complications (p<0.001), the Mann-Whitney test was carried out to compare the two groups.

**Table 2:** Comparison of age distribution, intra- operative and post-operative complications amongst the participants

**Sig.**	**Z**	**Mean ranks**	**Groups**	
0.91	-0.11	21.74	Age	Inner ear anomaly
		27.97	Intra- operative complications	
0.001	-3.29	19.32	Post- operative complications	
		21.34	Age	Normal
0.16	-1.39	17.10	Intra- operative complications	
		22.98	Post- operative complications	

According to the result of the Mann-Whitney test that is shown in Table 2, the two group's mean age was almost the same and the Mann-Whitney test showed no significant difference between the two groups (P =0.91). Concerning the descriptive statistics of the patients in the case and control groups, patients with inner ear anomaly, who experienced cerebrospinal fluid leakage that was controlled and sealed during the surgery were far more than the other group. The Mann-Whitney test result of Table 2 indicated the significance of the difference between the two groups with respect to intra-operative complications (P<0.001). Except for gusher, no major intra- operative complications, such as severe infection, facial nerve paralysis, electrode removal, and immediate or delayed prosthetic displacement were observed in the case or control group. Of note, none of the patients developed meningitis, and no major side effects were observed in the control group. The NRT conducted immediately after the operation in the case group was not recorded for 12 patients (70.58%), but it was observed in 5 patients (29.42%). In the control group, the NRT waves were recorded for all patients (P<0.05). Examination of the electrode placement showed that in both the control and case groups, the entire length of the electrode was placed in the cochlea. Finally, no significant post-operative complications in the case and control groups was observed (P>0.001).

## Discussion

In the present study, complications during or after cochlear implant surgery amongst patients with various internal ear anomalies were examined and compared with a control group. Results indicated the significance of the difference between the two groups in terms of intra-operative complications. However, no significant post-operative complications in both groups was observed. What is more, the minor side effects were limited and not significantly different. In a study by Ho Ahn, 80 out of the 388 patients had various internal ear anomalies (8). Immediate complications were defined as those occurring within 1 week of implantation and delayed complications as those occurring after 1 week. Minor and major complications were considered by severity requiring further management. 20 cases (25.0%) had postoperative complications, including facial nerve palsy, recurrent meningitis, device failure, and cerebrospinal fluid (CSF) leakage. Total of 3.8% of the patients required re-implantation. Minor complications were delayed, including middle ear infection with discharge (8). However, amongst the mentioned complications, only CSF leakage was seen in our study participants.

 Amongst 315 patients who had undergone cochlear implants in a study conducted by Buchman in 2004, 8.5% had internal ear anomaly, which was detected via radiographs (11). The Buchman’s study main focus was on auditory perception performance of cochlear implantation in children with inner ear anomaly. Results indicated various degrees of auditory benefits from this intervention. The different types of inner ear malformations might have different prognoses for auditory performance. In the Buchman et al., study on the patients with inner ear anomaly who underwent cochlear implant surgery, CSF gusher was observed in 21% (6 cases), and only 3 patients needed reoperation. Also, one patient underwent surgery to remove the electrode from the site (Mondini), one underwent facial nerve stimulation (along with facial nerve anomaly), and one patient underwent coronary artery bypass grafting due to incorrect placement of the electrode. Facial nerve stimulation occurred in 3 cases (11). It is necessary to remind that in both the case and control groups of our study, the entire length of the electrode was placed in the cochlea. Likewise, in a study by Buchman et al., in 89% of cochlear implanted cases, the entire length of the electrode was placed, and in two cases (11%) it was accompanied by incomplete placement (11).In the present study, Mondini was the most common anomaly amongst patients in the case group (47.1%). CSF gusher was observed in 64.7% of the patients in the case group, and 20% in the control group. The same pattern was also observed in 66.77% of the patients in a study conducted by Khalessi et al., in 2004 (12). In that study, 6 patients with internal ear anomalies underwent cochlear implant surgery, out of which one had incomplete partition, another had a common cavity, one had a narrow inner ear canal, and three had large vestibular aqueduct (LVA) (12). 

Facial nerve was anomalous in 2 cases. CSF gusher, which was controlled with packing the cochleostomy site was occurred in 4 patients. In all cases, the full length of electrode array was inserted, except one with Mondini's dysplasia who underwent reoperation on the opposite ear because of the insertion failure in the first operation. No other surgical complications were occurred.

In a study by Hashemi et al., the overall complication rate was 14%, and the major complications were prosthesis failure, incorrect electrode placement, and extensive hematoma that were resolved with surgical intervention (13). In a study by Natalie Loundon et al., the overall rate of complications was 9.9%, including major ones at 5.5%, and minors at 4.4%. The side effects were delayed in 65.1% of patients (14). As the main objective of cochlear implantation is to help children improve their auditory perception performance, various studies were carried out in this regard. A good case to illustrate is the study by Karamert, R. et al (2021) that exemplify the possibility of communication improvement amongst the children with inner ear malformations. Patients with enlarged vestibular aqueduct (EVA), had the best performance in terms of auditory-verbal skills. While the Patients with inner ear mal-formations such as incomplete partition Type 2, and cochlear hypoplasia type 2 scored poorly in comparison to patients with normal cochleae (10). This is an important issue, which was not taken into consideration in our study. Thus, along with the findings in the present study, which confirms the correlation between internal ear anomalies and intra-operative complications, we suggest further study by considering and overcoming its two major limitations, including relatively small sample size, especially in the case group, in addition to evaluating auditory perception and speech intelligibility of the cochlear implanted children with inner ear anomalies. 

It is mainly because of the fact that failure to record NRT waves in 12 patients (70.5%) in the case group might be a negative predictor of auditory and speech perception performance in implanted children. 

## Conclusion

The two groups were significantly different in terms of intra-operative complications. But, there was no significant post-operative complications in both groups. Despite problems and limitations in cochlear implantation of the patients with inner ear malformations, the cochlear implant surgery can be recommended with more confidence in this group of patients.
